# Supplementary material for: Antibiotic Breakdown by Susceptible Bacteria Enhances the Establishment of β-Lactam Resistant Mutants
Source: Front Microbiol. 2021 Aug 19;12:698970. doi: 10.3389/fmicb.2021.698970 (PMC8417073; doi:10.3389/fmicb.2021.698970)
Supplement: Supplementary Material 1 — ANOVAs and post hoc tests of the Interaction experiments. [file Data_Sheet_1.pdf]

Supplementary Material

1 ANOVAs and posthoc tests of the Interaction experiments

1.1 Interaction experiment 1

Table S1: Effect of Ancestor presence on the establishment of three tested mutant strains presented in Figure 1 (main article). Results of the two-way ANOVAs run per strain for effects of added Ancestor cells (*anc*), CTX concentration (*ctx*) and Ancestor cells by CTX interaction (*anc:ctx*), and results of Tukey's HSD tests. F-statistics of the ANOVAs are shown across all treatment comparisons per strain; results of Tukey's HSD tests are shown only for comparisons between the number of added Ancestor cells within each CTX concentration (per strain). Significant p-values are depicted in bold. All data were *sqrt* transformed. *N* = 15, except for the 0.14 µg/ml CTX (Single mutant) treatment where *n* = 14 per tested number of Ancestor cells.

**Two-way ANOVA**	**Strain**	**CTX concentration (µg/ml)**	**Number of Ancestor cells**		
			0 vs. 10^3	*0 vs. 10^6*	*10^3 vs. 10^6*
anc $F_{2,149}$=100, **p<0.001**<br>*ctx* $F_{1,588}$=789, **p<0.001**<br>*anc:ctx* $F_{2,23}$=15.6, **p<0.001**	Single mutant	0.14	**<0.001**	**<0.001**	0.65
		0.20	**<0.05**	**<0.001**	**<0.001**
anc $F_{2,25}$=16.3, **p<0.001**<br>*ctx* $F_{1,25}$=32.2, **p<0.001**<br>*anc:ctx* $F_{2,2}$=1.2, p=0.30	Double mutant	1.12	0.72	**<0.001**	**<0.05**
		1.28	0.99	0.08	**<0.05**
anc $F_{2,18}$=16.2, **p<0.001**<br>*ctx* $F_{1,19}$=33.0, **p<0.001**<br>*anc:ctx* $F_{2,0.23}$=0.2, p=0.81	Triple mutant	1.44	0.78	**<0.01**	0.15
		1.76	0.24	**<0.001**	0.34

1.2 Interaction experiment 2

Table S2: Effect of alive or heat-killed Ancestor or Single mutant presence on the establishment of the Triple mutant. Results of a two-way ANOVA for effects of added background population (*pop*; cf. Figure 2 in the main text), CTX concentration (*ctx*) and background population by CTX interaction (*pop:ctx*), and results of the posthoc Tukey's HSD test. F-statistics of the ANOVA are shown across all treatment comparisons; results of Tukey's HSD test are shown only for comparisons between background populations within CTX concentrations. Significant p-values are depicted in bold; *n* = 15.

Two-way ANOVA	Strain/population combinations	CTX concentration (µg/ml)	
		1.44	*1.76*
pop $F_{4,40375}$=425.7, **p<0.001** *ctx* $F_{2,55998}$=1180.8, **p<0.001** *pop:ctx* $F_{4,553}$=5.84, **p<0.001**	*Triple mutant vs. heat-killed Ancestor*	0.99	0.92
	Triple mutant vs. heat-killed Single mutant	0.99	0.99
	Triple mutant vs. alive Ancestor	**<0.001**	**<0.001**
	Triple mutant vs. alive Single mutant	**<0.001**	**<0.001**
	Heat-killed Ancestor vs. heat-killed Single mutant	0.94	0.99
	Heat-killed Ancestor vs. alive Ancestor	**<0.001**	**<0.001**
	Heat-killed Ancestor vs. alive Single mutant	**<0.001**	**<0.001**
	Heat-killed Single mutant vs. alive Ancestor	**<0.001**	**<0.001**
	Heat-killed Single mutant vs. alive Single mutant	**<0.001**	**<0.001**
	Alive Ancestor vs. alive Single mutant	**<0.001**	**<0.001**

2 Determining establishment probability and the corresponding concentration

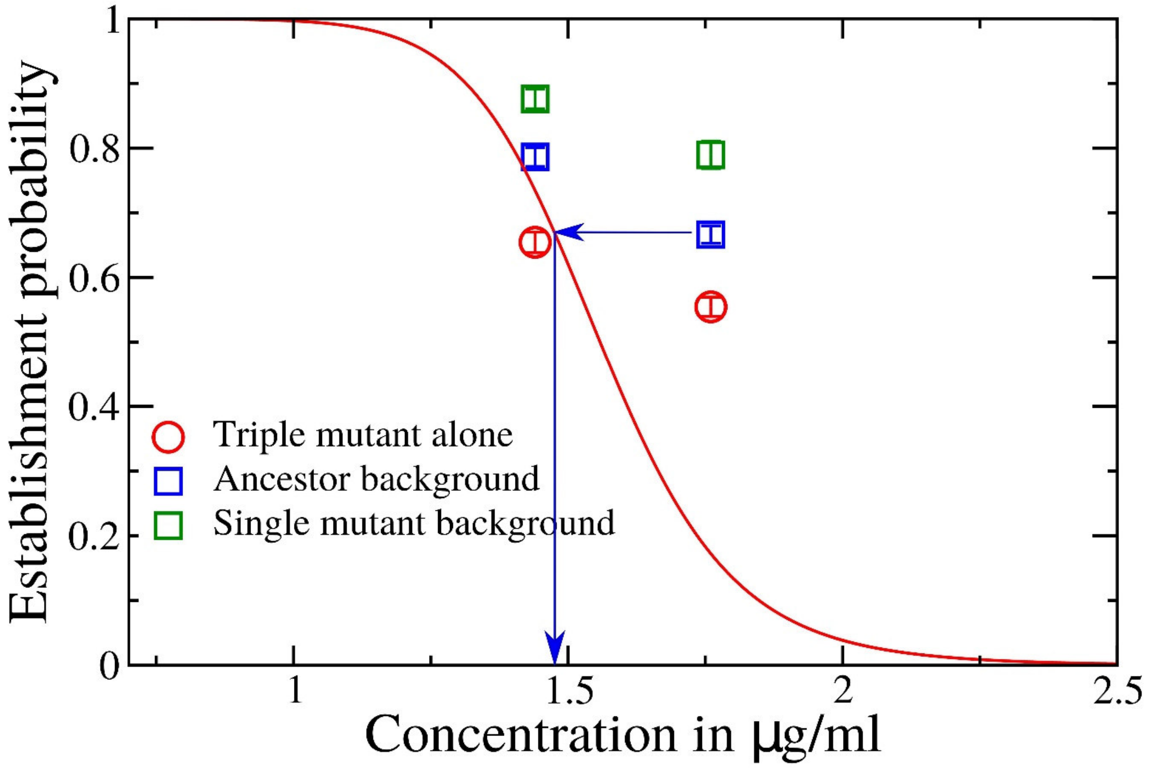

Figure S1: Inferred establishment probability of the Triple mutant in the absence and presence of Ancestor and Single mutant background cells. The establishment probabilities were obtained as the ratio of the mean CFU in the presence of 1.44 or 1.76 µg/ml CTX to that in the absence. The solid curve is obtained from a different study performed under the same conditions (Saebelfeld and Das et al., unpublished), but with a larger set of CTX concentrations. The solid red curve was determined by fitting a Hill function $p_e = \frac{1}{1+\left(\frac{x}{x_0}\right)^n}$; curve fitting yielded $n = 12.9$ ($\pm 11\%$) and $x_0 = 1.56$ ($\pm 2.0\%$) µg/ml. The estimate of the decreased concentration was done using this red curve; as an example, one may estimate the new, reduced concentration in the presence of the Ancestor at 1.76 µg/ml by following the blue arrows. The error estimates for reduction were performed by standard error propagation under the assumption of linearity. The discrepancy between the red symbols and the full curve reflects experimental uncertainties and inherent limitations of the model and provides a rough measure of the accuracy of the inferred concentration changes.

3 Expected CTX reduction in the bioassay

To understand why the CTX reduction in the bioassay by the Ancestor compared to the Single mutant is unexpectedly high, we begin with a simple calculation based on chemical kinetics. Let the concentration of antibiotic in the periplasmic space be B_{in}, and the enzyme concentration there be E. Then, the rate of reduction of concentration in the cell is

$$-\frac{dB_{in}}{dt} = \frac{k_{cat}}{k_M} E B_{in}. \tag{1}$$

For the Single mutant, we have $k_{cat}/k_M = 1.3 \times 10^5\ s^{-1}\ M^{-1}$ (Salverda et al., 2011). The enzyme concentration is 168 molecules per cell (Suvorov et al. 2007 found that about 12,600 molecules are expressed from about 75 plasmid copies, leading to the estimate of 168 molecules per gene copy). A cell volume is taken to be about 1 μm^3 (Kubitschek, 1969; Wang et al., 2008), and the periplasmic space volume is 0.3 μm^3 (Stock et al., 1977). Therefore the enzyme concentration is $E = 0.93$ µM. If the antibiotic concentration within the periplasmic space is the same as the initial concentration in the medium, 1.6 µg/ml (which is the average of the two highest concentrations tested in the plating experiments) or 3.6 µM (using the molecular weight 445 g/mol and the volume of periplasmic space); then the net reduction rate in the periplasmic space is computed from above to be 0.19 µg/ml per second per cell or 76 molecules per second per cell. Assuming a constant number of 2×10^8 cells and 3 hours incubation, one predicts a net reduction of 0.12 µg/ml in the medium, i.e. 7.5%. Similarly, for the Ancestor, $k_{cat}/k_M = 10^3\ s^{-1}$ (Salverda et al., 2011), and the reduction is predicted to be 9.2×10^{-4} µg/ml or 0.06%.

4 Dynamical model

We adopt a dynamical model from Geyrhofer and Brenner (2020). We define N as the number of cells in the medium, $B_{in}$and B_{out} as the antibiotic concentration inside and outside the cell respectively, and E_{in} and E_{out} as the concentration of enzyme inside and outside the cell, respectively. Then the system is described by the following equations

$$\frac{dN}{dt} = \alpha_e(B_{in})N$$

$$\frac{dB_{in}}{dt} = -\varepsilon E_{in} B_{in} + \sigma_B (B_{out} - B_{in})$$

$$\frac{dB_{out}}{dt} = \eta \sigma_B N (B_{in} - B_{out}) - \varepsilon E_{out} B_{out} \tag{2}$$

$$\frac{dE_{in}}{dt} = \rho + \sigma_E (E_{out} - E_{in}) \tag{3}$$

$$\frac{dE_{out}}{dt} = \eta N \sigma_E (E_{in} - E_{out}),$$

where we take the growth rate α_e to have the empirical form (Geyrhofer and Brenner, 2020)

$$\alpha_e(B_{in}) = \alpha \frac{1-\left(\frac{B_{in}}{\mu}\right)^{\kappa}}{1+\frac{1}{\gamma}\left(\frac{B_{in}}{\mu}\right)^{\kappa}}. \tag{4}$$

The terms containing σ_B and σ_E correspond respectively to the fluxes of antibiotic and enzyme between a cell and the medium, and the quadratic terms containing ε are the breakdown rates of antibiotic by the enzyme. Note that the breakdown is taken to occur both within and outside the cell.

We take $\gamma = 1$ (Geyrhofer and Brenner, 2020) and $\kappa = 2$ (the precise value does not matter); α is the growth rate in the absence of antibiotics, which we take to correspond to a division time of 30 minutes. The parameter μ is the MIC of a cell in the absence of any enzyme activity, such as the cells of the Wildtype strain without the TEM allele in the bioassay. We do not know this value, but we can establish upper and lower bounds and estimate the single-cell MIC as the geometric mean of the two. It should be less than the MIC of the Ancestor, which is 0.04 µg/ml (based on single-cell MICs run in triplicates with 2-fold increases in CTX concentration and 4-fold decreases in inoculum size starting from 10^5 cells; data not shown), or about 52 molecules in 1 μm^3, which is the cell volume. We do not expect the MIC to be less than 1 molecule per cell, establishing the lower bound. The geometric mean gives us the estimated MIC, $\mu = 7.2$ molecules per μm^3. The volume separation factor η is the ratio of the volume of the periplasmic space (about 30% of the cell volume (Stock et al., 1977), which is 1 μm^3) to the volume of the medium, which is 1 ml. This comes out as $\eta = 3 \times 10^{-13}$. The reaction rate constant $\varepsilon = \frac{k_{cat}}{k_M}$ is $0.78\ (\mu m^3 hr)^{-1}$for the Single mutant, and $6.0 \times 10^{-3}\ (\mu m^3 hr)^{-1}$ for the Ancestor (Salverda et al., 2011). The synthesis rate of the enzyme is ρ (Eq. 3), whereas σ_E is the rate constant for enzyme secretion from the cells. When the enzyme concentration outside the cell is low, the steady-state concentration of the enzyme in the cells is given $E_{in}^{ss} = \frac{\rho}{\sigma_E}$. We use the value of 168 molecules per cell (see the previous subsection) for E_{in}^{ss} , and this fixes the ratio of ρ and σ_E. Determining one of them fixes the other. The rate constant for antibiotic flux through the cell is σ_B.

We are finally left with two undetermined parameters, σ_B and σ_E, and we need to explore this parameter space to understand the amount of reduction achieved. Figure S2 shows the simulation results for this model, over large ranges of the two parameters σ_B and σ_E. The solid lines belong to the Single mutant. The parameter regime we are interested in should produce a breakdown of a few tens of per cent, and growth in cell number by a factor of about 10 or less (corresponding to a generation time of about 1 hour and a similar lag time preceding growth; based on previous observations with the tested strains under similar conditions, data not shown). By these criteria, the relevant ranges are seen to be σ_B about $1{\sim}3\ h^{-1}$, and σ_E in the range $1{\sim}10\ h^{-1}$. (A more detailed numerical exploration shows that the values $\sigma_E = 2.81\ h^{-1}$; $\sigma_B = 1\ h^{-1}$ produce a reduction of approximately 27% and a final cell population 3.6 times higher than the initial value, close to the value 4 we would get with one hour lag and one hour doubling time). The precise parameter and reduction values are unimportant; we are interested in whether the Ancestor cells have the same order-of-magnitude reduction in the relevant parameter range. The dashed lines in Figure S2 show the reduction of antibiotic by the Ancestor, and the reduction is about 2 to 3 orders of magnitude lower than that due to the Single mutant in our region of interest. Specifically, for $\sigma_E = 2.81\ h^{-1}$; $\sigma_B = 1\ h^{-1}$, the reduction is 0.062%, which is far too small. The experimental observation is therefore not fully explained. However, the blue dashed-dotted line includes the effect of 100% filamentation among the Ancestor cells, and it exhibits a much higher reduction - close to the actual amount of reduction observed.

Filamentation is numerically simulated as follows. We assume two kinds of cells: non-filamenting cells with biomass N_0 (in units of single cell biomass), and filamenting cells with biomass N_f. The non-filamenting cells grow at rate $\alpha_e(B_{in})$ and transition to filamentation at rate r. The filamentous cells do not divide but their biomass keeps growing at the uninhibited rate α. The equations describing the dynamics are

$$\frac{dN_0}{dt} = \alpha_e(B_{in})N_0 - rN_0 \quad (5)$$

$$\frac{dN_f}{dt} = \alpha N_f + rN_0. \quad (6)$$

The total cell biomass is $N = N_0 + N_f$. This value of N is now used in the dynamical equations for $B_{in}, B_{out}, E_{in}, E_{out}$. The normalized filamentation rate given in the main text is calculated as $f = \frac{r}{r+\alpha} = \frac{r/\alpha}{\left(\frac{r}{\alpha}\right)+1}$. This definition makes the quantity f dimensionless and restricts it to the range $0 \leq f \leq 1$. It can be interpreted as the probability that a cell filaments rather than divides when the filamentation rate is r and the growth rate of non-filamentous cells is the drugless rate α.

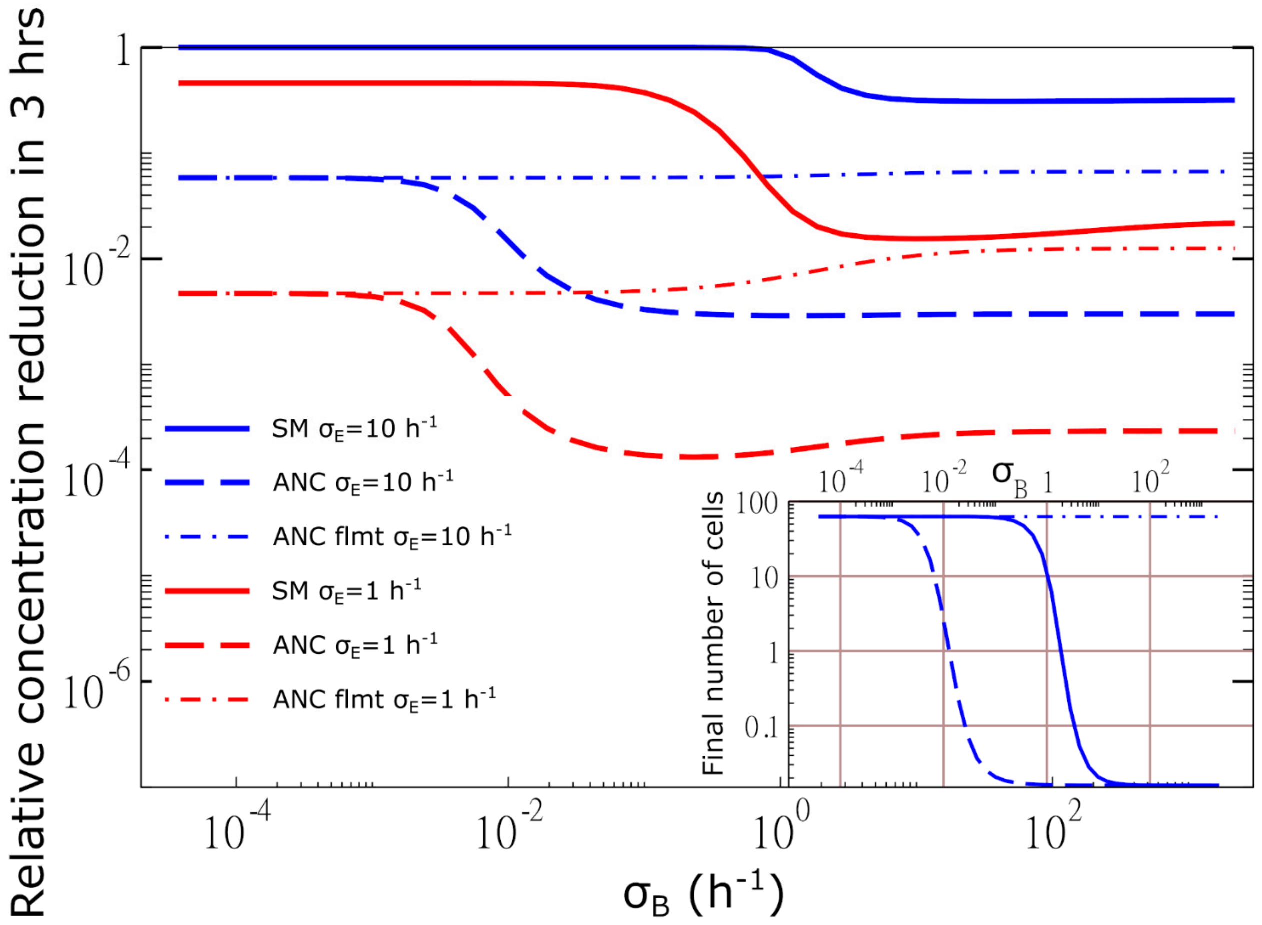

Figure S2: Simulation results for antibiotic concentration reduction in liquid. Solid lines are for the Single mutant (SM), dashed lines for Ancestor (ANC) without filamentation. The dash-dotted lines are for the Ancestor with filamentation and two different enzyme transport rates. The inset shows the number of cells after 3 hours.

5 References

Geyrhofer, L., and Brenner, N. (2020). Coexistence and cooperation in structured habitats. *BMC Ecol.* 20, 1–15. doi:10.1186/s12898-020-00281-y.

Kubitschek, H. E. (1969). Growth during the bacterial cell cycle: analysis of cell size distribution. *Biophys. J.* 9, 792–809. doi:10.1016/S0006-3495(69)86418-0.

Salverda, M. L. M., Dellus, E., Gorter, F. A., Debets, A. J. M., van der Oost, J., Hoekstra, R. F., et al. (2011). Initial mutations direct alternative pathways of protein evolution. *PLoS Genet.* 7. doi:10.1371/journal.pgen.1001321.

Stock, J. B., Rauch, B., and Roseman, S. (1977). Periplasmic space in *Salmonella typhimurium* and *Escherichia coli*. *J. Biol. Chem.* 252, 7850–7861.

Suvorov, M., Vakulenko, S. B., and Mobashery, S. (2007). Cytoplasmic-membrane anchoring of a class A β-lactamase and its capacity in manifesting antibiotic resistance. *Antimicrob. Agents Chemother.* 51, 2937–2942. doi:10.1128/AAC.00011-07.

Wang, Y., Hammes, F., Düggelin, M., and Egli, T. (2008). Influence of size, shape, and flexibility on bacterial passage through micropore membrane filters. *Environ. Sci. Technol.* 42, 6749–6754. doi:10.1021/es800720n.
